# Endometritis and *In Vitro* PGE_2_ Challenge Modify Properties of Cattle Endometrial Mesenchymal Stem Cells and Their Transcriptomic Profile

**DOI:** 10.1155/2017/4297639

**Published:** 2017-10-29

**Authors:** Evelyn Lara, Alejandra Velásquez, Joel Cabezas, Nathaly Rivera, Paulina Pacha, Lleretny Rodríguez-Alvarez, Fernando Saravia, Fidel Ovidio Castro

**Affiliations:** Department of Animal Science, Faculty of Veterinary Sciences, Universidad de Concepción, Chillán, Chile

## Abstract

Mesenchymal stem cells (MSCs) were isolated and characterized from postpartum bovine endometrium of animals with subclinical (*n* = 5) and clinical endometritis (*n* = 3) and healthy puerperal females (*n* = 5). Cells isolated displayed mean morphological features of MSCs and underwent osteogenic, chondrogenic, and adipogenic differentiation after induction (healthy and subclinical). Cells from cows with clinical endometritis did not undergo adipogenic differentiation. All cells expressed mRNAs for selected MSC markers. Endometrial MSCs were challenged in vitro with PGE_2_ at concentrations of 0, 1, 3, and 10 *μ*M, and their global transcriptomic profile was studied. Overall, 1127 genes were differentially expressed between unchallenged cells and cells treated with PGE_2_ at all concentrations (763 up- and 364 downregulated, fold change > 2, and *P* < 0.05). The pathways affected the most by the PGE_2_ challenge were immune response, angiogenesis, and cell proliferation. In conclusion, we demonstrated that healthy puerperal bovine endometrium contains MSCs and that endometritis modifies and limits some functional characteristics of these cells, such as their ability to proceed to adipogenic differentiation. Also, PGE_2_, an inflammatory mediator of endometritis, modifies the transcriptomic profile of endometrial MSCs. A similar situation may occur during inflammation associated with endometritis, therefore affecting the main properties of endometrial MSCs.

## 1. Introduction

During the female reproductive lifespan, the bovine endometrium periodically undergoes morphological and functional modifications [1]. These are coupled with variations in the gene expression pattern involved in endometrial remodeling, the regulation of angiogenesis, regulation of invasive growth, cell adhesion, and embryo feeding [2]. The high and continuous cell regeneration of the endometrium has been ascribed to the presence of resident progenitor/stem cells in the uterus, which maintain the cellular production and quickly restore the necessary tissue homeostasis to support a gestation [3]. It is suggested that these cells may also facilitate endometrium regeneration that takes place immediately after parturition [4]. Postpartum is one of the main periods of renovation, repair, and endometrial regeneration, leading to quick uterine involution from day 8 to day 43 postpartum in cattle [5]. This involution may be affected by the exposition of the uterus to multiple bacterial pathogens that are frequently observed in the postpartum period and can generate significant damage to endometrial tissue [6].

Pathogenic bacteria affect 90 to 100% of dairy cows after parturition. They are relatively eliminated rapidly in most cases (around 70%); however, a persistent inflammatory response occurs in the remaining 30% [7, 8]. Endometritis is the inflammation of the endometrium and is classified as subclinical and clinical, frequently causing infertility due to damage to the tissue and disruption of ovarian cyclic activity [9].

Postpartum inflammation of the uterus involves an immune and inflammatory local response encompassing a number of molecules; the inappropriate balance between pro and anti-inflammatory cytokines determines the continuity, duration, and onset of the inflammatory disease [7]. Prostaglandin E2 (PGE_2_) plays a critical role in various aspects of the inflammatory response by regulating the production of various interleukins (ILs) and tumor necrosis factor alpha (TNF-*α*), provoking potent immunosuppressive properties that contribute to the resolution of acute inflammation, tissue regeneration, and the return to homeostasis [10]. Endometrial cells from cows with clinical endometritis have been found to secrete significantly higher levels of PGE_2_ in comparison to healthy endometrial and subclinical endometritis groups; in cows with subclinical endometritis, the said levels were much higher in the group diagnosed with >18% of polymorph nuclear cells (PMNs) than the group with >5% of PMN [11]. It is suggested that PGE_2_ concentrations in the uterine fluid are related to the endometritis degree and uterine endotoxin concentrations, which induce and activate the specific production of this molecule via COX2 [12]. Similar to many other mediators of cellular responses, PGE_2_ is multifunctional and may play a role in improving homing, survival, and proliferation of mesenchymal stem cells (MSCs) [13].

MSCs are present in the endometrium of several species including humans, mice, pigs, and ruminants [14–18]. Our group was able to isolate and characterize these cells in cattle endometrium for the first time throughout all the stages of the estrus cycle in healthy bovine endometrium [19, 20]. Taking into consideration the crucial role of PGE_2_ in endometritis-mediated inflammation as well as in MSC rescue as described earlier and the presence of MSCs in the endometrium of healthy cows, we postulated the hypothesis that pathological inflammation of the uterus as in endometritis affects the presence and functionality of such endometrial MSCs. This study aimed to investigate the presence of MSCs in the puerperal bovine endometrium, in both healthy cows and those with subclinical and clinical endometritis, as well as to evaluate their essential intrinsic biological attributes. We hypothesize that PGE_2_ may play a pivotal role in the activation and recruitment of putative MSC in the endometrium of these cows and/or in healthy cows. To assess this, we first studied and characterized putative MSCs in puerperal endometrium of healthy and endometritis-ill cows. In the second set of experiments, bovine endometrial MSCs previously isolated and established in the laboratory were challenged with different PGE_2_ concentrations. Subsequently, their transcriptomic changes were studied in an attempt to dissect the actual role of PGE_2_ in endometrial MSC biology in a cow animal model.

## 2. Materials and Methods

All the experimental procedures involving animal handling and/or sampling were approved by the Bioethics Committee of the Universidad de Concepción, Chile, under the approval number CE-6-2010 and were conducted according to the regulations for animal well-being of the Faculty of Veterinary Sciences of the same university.

### 2.1. Experiment 1: Isolation and Characterization of Putative MSCs in Postpartum Bovine Endometrium

All unspecified reagents were obtained from Sigma-Aldrich (St. Louis, MO, USA).

#### 2.1.1. Uterine Samples

Endometrial biopsies were collected between 20 to 60 days postpartum from Holstein cows in a productive herd and in postpartum healthy endometritis (PPHE, *n* = 5), with subclinical (PPSE, *n* = 5) and clinical (PPCE, *n* = 3) endometritis. Cows were aged between 3 and 6 years. Sample classification was based on a veterinary clinical examination of the endometrium and cytological analysis determining the percentage of PMN, following the criteria of LeBlanc et al. [21] and Sheldon et al. [22].

#### 2.1.2. Cell Isolation

Tissue samples were digested with sterile 1 mg/ml collagenase type I in phosphate-buffered saline (PBS) solution for 2 h at 37°C and were centrifuged at 550 g for 10 min. Cells were seeded at 0.5 × 10^5^ cells/ml density in standard medium of DMEM-F12 with 10% fetal calf serum (FCS) supplemented with 1% antibiotic-antimycotic (AAM) solution, 1 mM sodium pyruvate, and 2 mM L-glutamine and were cultured in 5% CO_2_, 39°C, and 100% humidity. To separate the epithelial cells from the stromal cells, the supernatant was removed approximately 18 hr after the first seeding and reseeded in the same way. The medium was changed every 2-3 days until cells reached confluence.

#### 2.1.3. Colony Formation

The cells were seeded in 60 mm plates at 30 cells/cm^2^ density in the same environmental and medium conditions as in the primary cell culture, changing the medium every 15 days. The experiments were performed in triplicate for all the cell lines. The plates were microscopically examined to make sure that each colony originated from a single cell. After 1 month, colonies were fixed with 2% paraformaldehyde (PFA) and stained with Giemsa to allow for clone visualization. Colonies containing more than 20 cells were counted. To evaluate the ability of cells to form colonies or clonal efficiency (CE), the following formula was used: (number of clonal cells/number of inoculated cells) × 100% [3]. Colonies were stained with alkaline phosphatase (AP) according to protocol (Vector® Red substrate; Vector Laboratories, Burlingame, CA, USA) with a positive AP staining, characterized by a red coloration and observed with digital inverted microscope fluorescence EVOS FL (Life Technologies, Carlsbad, CA, USA).

#### 2.1.4. Cell Proliferation

Cells were seeded in triplicate in 60 mm plates at 2000 cells/cm^2^ density and cultured under the same environmental and medium conditions as in the primary cell culture. Cell doubling time (CDT) was calculated using the following formulas: CD = ln (Nf/Ni)/ln 2, with Nf being the final and Ni the initial number of cells and DT = CT/CD, where DT is the CDT and CD is the cell doubling number, with CT being the cell culture time [23].

#### 2.1.5. *In Vitro* Differentiation into Mesodermal Derivatives

Cells in passage 3 were seeded at 4 × 10^4^ cells/per well in six-well dishes in triplicate until they reached 90% confluence and then changed to differentiation media (DM). Differentiation media were based on DMEM low glucose with 10% FCS and supplemented to induce differentiation into chondrogenic lineage (CL), with 100 nM dexamethasone (Dex), 35 *μ*g/ml vitamin C, 1x insulin selenium transferrin (ITS), and osteogenic linage (OL) with 50.9 *μ*M Dex, 10 mM *β*-glycerophosphate, 0.1 mM vitamin C, and adipogenic linage (AL) with 1 *μ*M Dex, 22 *μ*g/ml 1x ITS, 0.25 mM 3-isobutyl-1methylxanthine (3-IBM), and 100 mM indometacin. Control time-mated cells were cultured in DMEM low glucose + 10% FCS, without inducers for exactly the same time periods as the experimental groups. In all cases, media were changed every 3 days. At days 0, 7, and 14, cells were fixed with 2% PFA and stained with Alcian blue for CL, alizarin red for OL, and Oil Red for AL to detect the expression of glycosaminoglycan, calcium deposition, or lipid vacuoles, respectively.

#### 2.1.6. Quantitative Real-Time Polymerase Chain Reaction (RT-qPCR)

At the time points mentioned above, the total RNA was extracted from the cells using Tri-reagent extraction according to the manufacturer's instructions. The quantity of RNA was measured using an Epoch Spectrophotometer System (Biotek, Bad Friedrichshall, Germany). The cDNA was transcribed from 200 *μ*g of the total RNA according to the manufacturer's instruction of a commercial M-MLV Reverse Transcriptase (RT) (Invitrogen). Gene expression analysis was performed by real-time PCR by means of the standard curve method using SYBR Green on an MX3000P real-time PCR device (Agilent, Santa Clara, CA, USA). In all qPCRs, beta-actin (ACTB) was used as an internal control.

Only PCR experiments with an efficiency within the range of 90–110% and with a correlation coefficient of at least 0.97 were used for gene expression analysis. Samples were run in triplicates. qPCR was performed with the primer sequences displayed in Table 1.

#### 2.1.7. Data Analysis

Statistical analysis of quantitative real-time PCRs, clonogenic, and proliferation assays was conducted using a nonparametric test (Kruskal Wallis), and the data was expressed as mean ± error. All statistical analyses were tested for *α* = 0.05.

### 2.2. Experiment 2: Gene Expression in Bovine Endometrial Putative MSCs Challenged with PGE_2_

All reagents otherwise expressed were from Thermo Fisher, Santiago de Chile, Chile.

#### 2.2.1. Cell Lines

Primary culture of putative MSCs from healthy cycling cows in late luteal phase was used (LLP1 and LLP4; [19]). Cells were seeded at 40000/cm^2^ in 12-well culture dishes and maintained and cultured in standard medium and under the same conditions as in the first experiment. Cells were allowed to reach 90% confluency and were further used in priming experiments.

#### 2.2.2. Priming with PGE_2_

The medium was removed from the wells, the cells were washed three times in warm PBS, and 2 ml of media containing three different concentrations of PGE_2_ was added per well in triplicate. The PGE_2_ (Cayman Chemical, Ann Arbor, Michigan, USA) was diluted in DMSO (pH 6.8 to avoid PGE_2_ degradation), and the final concentrations were 1, 3, and 10 *μ*M. As negative controls, cells from the same origin were used in triplicate under the same culture conditions but not primed with PGE_2_. After 28 hours, the supernatant was collected and frozen at −80°C for future use, and the cells were scrapped and subjected to RNA extraction for further processing.

#### 2.2.3. RNA Extraction and Synthesis of Complementary RNA

The total RNA was isolated and quantified as previously described (experiment 1). The RNA integrity number (RIN) was determined using TapeStation 2200 system (Agilent Technologies©, Santa Clara, CA, USA). Only samples with RIN > 8.5 were selected for analysis. The RNA Spike-In kit (Agilent Technologies©, Santa Clara, CA, USA) was used as external control. Spike A Mix with cyanine-3 was used to label the samples (primed and nonprimed cells), and Spike B Mix with cyanine-5 was used to label a reference (bovine fibroblast RNA). The Agilent Low Input Quick Amp Labeling kit (Agilent Technologies©, Santa Clara, CA, USA) was used to generate complementary RNA (cRNA) with a sample input of 100 ng total RNA. The cRNAs were then purified using EZNA Total RNA kit I according to the manufacturer's instructions and quantified using Epoch Spectrophotometer System (Biotek©, Bad Friedrichshall, Germany). For cRNA, pools of the three replicates of each three doses and of the nonprimed cells were used.

#### 2.2.4. Hybridization, Washing, and Scanning of Microarrays

The bovine (V2) gene expression microarray 4 × 44 (Agilent Technologies, USA) was used for differential gene expression analysis. Hybridization mixtures were prepared using the Hi-RPM Gene Expression Hybridization kit (Agilent Technologies, USA). According to Agilent protocol, for a 4 × 44 K microarray, 825 ng of each cRNAs was used. The Cy-3 and Cy-5–labeled cRNA samples were mixed, hybridized, and added to the microarray slide. The slide chamber was assembled and placed in a rotisserie hybridization oven and rotated at 10 rpm in 65°C for 17 hours. The array slides were washed using Gene Expression Wash Buffer Kit. After being washed, the slides were scanned using Agilent's SureScan Microarray Scanner at settings recommended for the 4 × 44 K array format. Images obtained after scanning were analyzed using Agilent Feature Extraction software v.10.5.1.1.

#### 2.2.5. Microarray Data Analysis

Data obtained were analyzed using GeneSpring 12.5 extraction software (Agilent Technologies, USA) in order to determine which genes were differentially expressed between the experimental groups in relation to the control nonprimed cells. Genes were determined to be differentially expressed if there was a greater than 2-fold change in upregulation or downregulation. Statistical analysis was carried out using an unpaired *t*-test, and a fold change with a cut-off value greater than 2 with a *P* value 0.05 was considered to indicate a statistically significant difference.

#### 2.2.6. Gene Ontology (GO) and Interaction Network of Differentially Expressed Gene Analysis

Gene ontology and pathway analysis were performed using Panther software 11.1 (University of Southern California, USA). Both analyses were performed based on the differentially expressed gene list with fold change greater than 2.0. For GO analysis, a corrected cut-off *P* value of 0.58 was used (minimal value to detect GO terms). Pathway analysis was based on WikiPathways database. The Panther software is able to detect potential connections between the selected genes and to classify them according to *P* values and the number of matched entities per pathway. From the total and the number of significantly differentially expressed genes with a fold change greater than or equal to 2.0, the gene interaction network was created with the GeneMania Prediction Server [24].

#### 2.2.7. qRT-PCR Analysis for Microarray Validation

To validate microarray data, the expression profile of the selected genes was analyzed using qRT-PCR. To do this, 13 genes were selected, ten differentially expressed between both groups (four upregulated [UQCRB, ADA, F3, and BPGM], six downregulated [CFB, SLC35A5, SCRN1, IGFBP3, LOC781494, and TFPI2]), and three equally expressed (BAX2, COX2, and IL-1). The purified RNAs were treated with 1 U of RNAse-free DNase I for genomic DNA digestion (Invitrogen, Carlsbead, CA, USA) in a 10 *μ*l reaction for 30 minutes at 37°C. The enzyme was heat inactivated (65°C for 10 min) in the presence of 25 mM EDTA (1 *μ*l). The mRNA was converted to complementary DNA and kept frozen at 20°C until use in PCR experiments. Gene expression analysis was performed by qRT-PCR using the ∆∆Ct method. For qRT-PCR, samples were loaded as duplicates (technical replicates). The primers used and PCR conditions for each gene are added in Table 1. In all qRT-PCRs, B-actin was used as a housekeeping gene.

#### 2.2.8. Statistical Analysis of qRT-PCR

This was performed using a Wilcoxon nonparametric test. Microarray validation and correlation with qRT-PCR were conducted employing Pearson's correlation test, with the log2 of the ratio of means. In all cases, significant differences were considered if *P* values were less than 0.05. InfoStat (Buenos Aires, Argentina) software was used.

## 3. Results

### 3.1. Experiment 1: Isolation and Characterization of Putative MSCs in Postpartum Bovine Endometrium

#### 3.1.1. Ability to Form Colonies and to Proliferate

**Figure 1:**
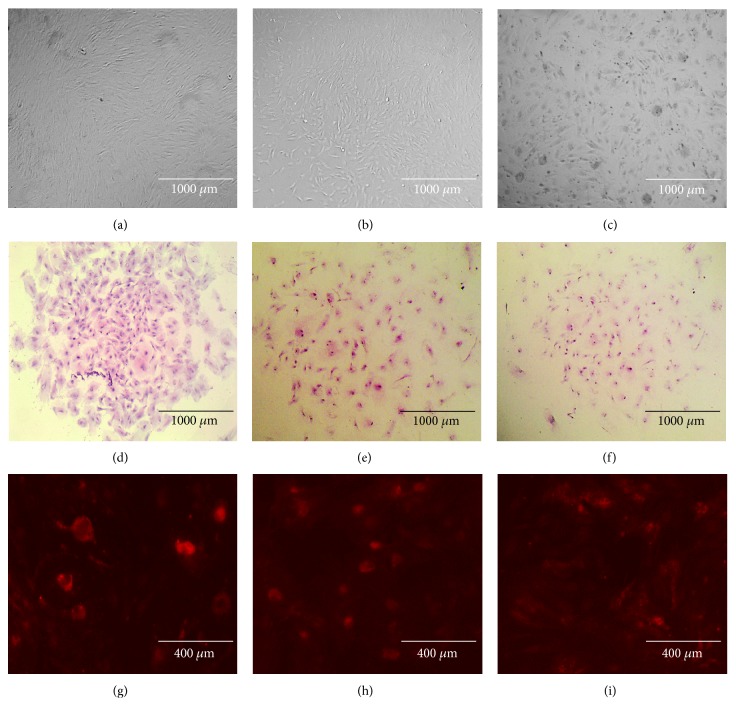
Representative images of the morphology and *in vitro* colony formation of the primary cell cultures derived from bovine endometrial tissue of a healthy animal (a, d, g) with subclinical endometritis (b, e, h) or clinical endometritis (c, f, i). (a, b, c) Normal fibroblast-like appearance at 40x. (d, e, f) Giemsa stain at 40x. (g, h, i) Alkaline phosphatase activity in a colony at 100x.

All isolated cells showed fibroblast-like morphology and adherence to plastic and, when seeded at low confluence, proliferated and produced colonies with a positive AP staining (Figure 1). The number of colonies was significantly higher in HPPE cells than in CPPE (5.42 ± 0.7 versus 0.83 ± 0.8 clones per dish, resp.), as well as cloning efficiency (CE of 0.64 ± 0.1% versus 0.08 ± 0.1). Meanwhile, the average cell doubling time (CDT) was significantly lower in HPPE than in CPPE, with a value of 30 ± 0.4 hours versus 41.97 ± 1.4 (Table 2). The HPPE cells filled the culture dishes in 5 to 7 days, whereas those SPPE or CPPE achieved full confluency at 8 to 10 days and 10 to 12, respectively.

#### 3.1.2. Multilineage *In Vitro* Differentiation

**Figure 2:**
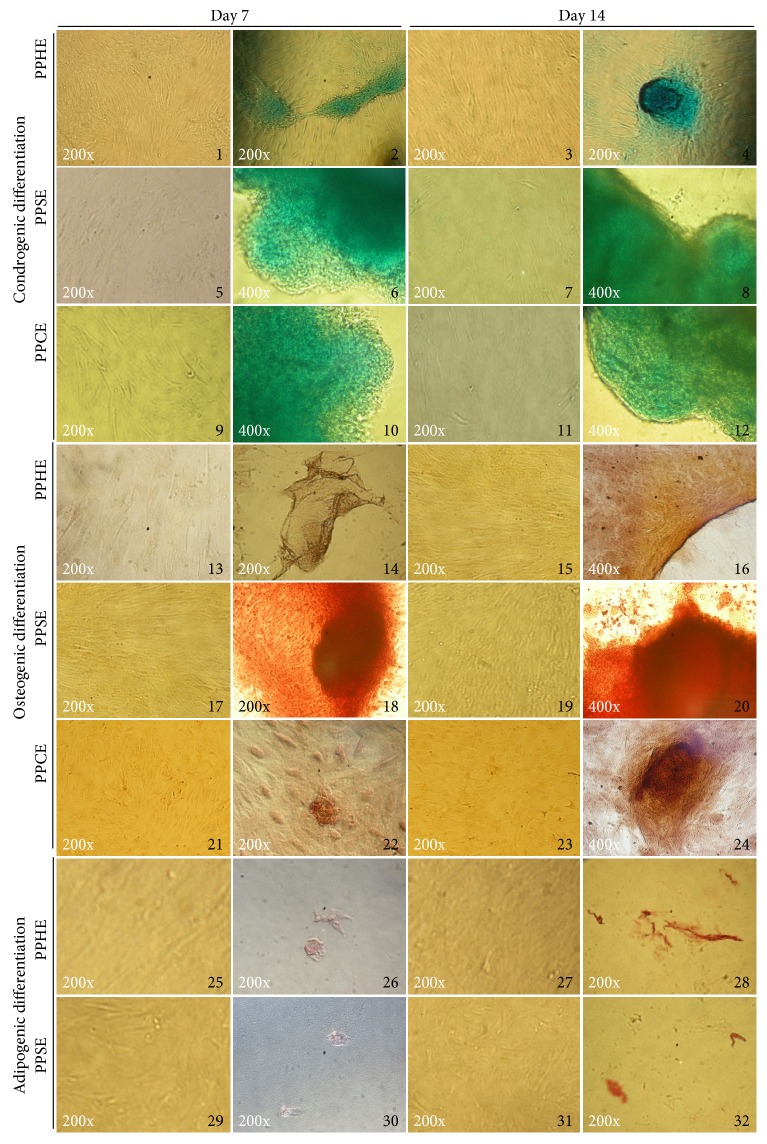
Representative images from the *in vitro* differentiation to chondrogenic (2, 4, 6, 8, 10, and 12), osteogenic (14, 16, 18, 20, 22, and 24), and adipogenic (26, 28, 30, and 32) lineages of cells from bovine postpartum healthy endometrium (PPHE) and subclinical (PPSE) and clinical (PPCE) endometritis at day 7 and 14. Noninduced cell controls (1, 3, 5, 7, 9, 11, 13, 15, 17, 19, 21, 23, 25, 27, 29, and 31).

Cells were responsive to differentiation stimuli upon induction to chondrogenic, osteogenic, and adipogenic lineages. Visually, the intensity of staining at fixed time points (day 7 or 14 of induction) was similar among the cell lines and we did not attempt to quantify the staining. However, adipogenic staining with Oil Red was detected only in healthy cows or in cows with subclinical endometritis. No staining was observed in noninduced stained controls (Figure 2).

#### 3.1.3. Expression of Gene and Protein Markers

**Figure 3:**
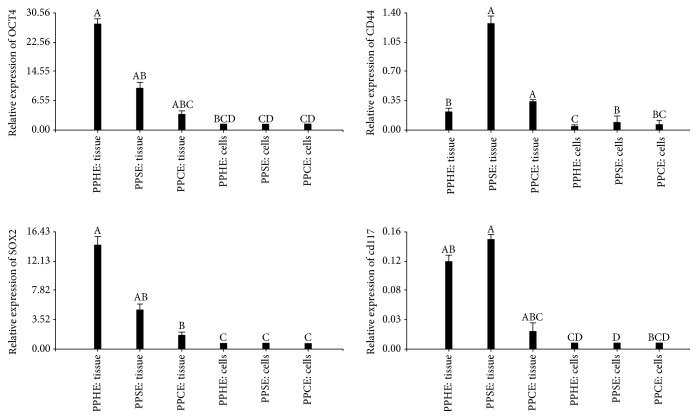
Reverse transcription and real-time PCR analysis of the expression of OCT4, SOX2, CD44, and CD117 markers in tissue and cells from primary culture from bovine postpartum healthy endometrium (PPHE) with subclinical (PPSE) or clinical (PPCE) endometritis (a, b). All PCRs were normalized against ACTB. Different letters show significative differences with *P* < 0.05.

**Figure 4:**
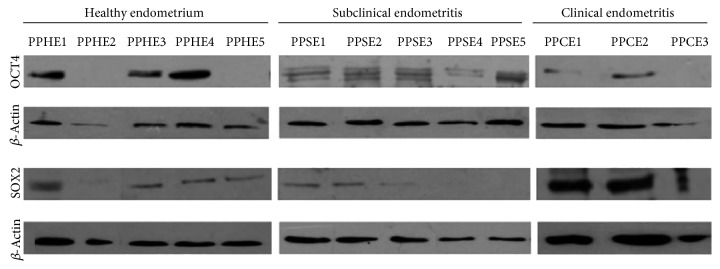
Expression of the OCT4 and SOX2 proteins through western blot in cells from bovine postpartum endometrial primary culture.

Tissue biopsies and cells isolated from the bovine endometrium and cultured *in vitro* expressed OCT4, SOX2, CD44, and c-KIT but not NANOG. OCT4 and SOX2 were expressed higher in tissues or in cells from healthy animals, and in general, the expression of all genes was stronger in tissues than in cells cultures derived from the said tissues (Figure 3). The presence of pluripotency markers OCT4 and SOX2 was confirmed in tissue by western blot, in most tissue samples for all pathological conditions of bovine endometrium (Figure 4).

### 3.2. Experiment 2: Gene Expression in Bovine Endometrial Putative MSCs Challenged with PGE_2_

#### 3.2.1. Differential Transcriptomic Analysis of Gene Expression

**Figure 5:**
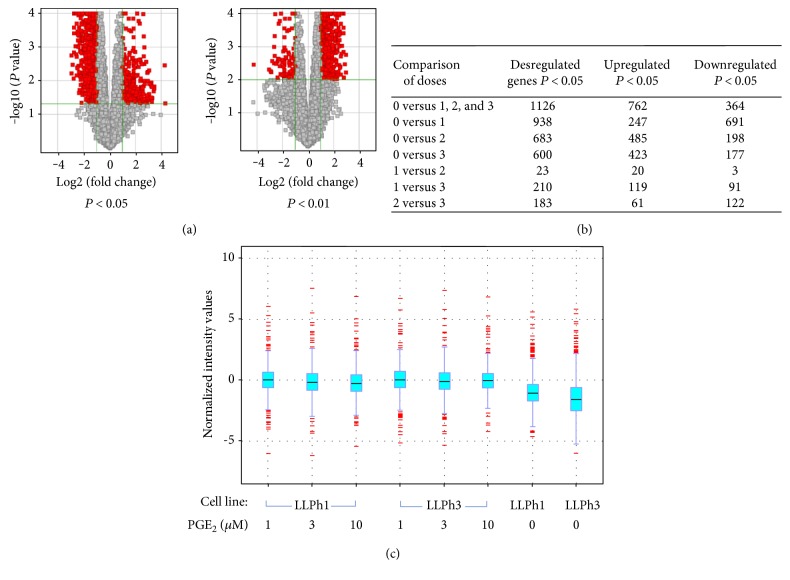
Volcano plot showing the distribution of differentially expressed genes in PGE_2_+ cells as a function of the selected *P* value. Left panel *P* < 0.05, right panel *P* < 0.01 (a). Details of differential response of endometrial MSCs to the challenge with PGE_2_ (b). Cat whiskers plot representing the distribution of normalized intensity values among the two cell lines (LLPh1 and LLPh3) and the four doses (0, 1, 3, and 10 *μ*M of PGE_2_) used. The amplitude of the intensity values is wider for PGE_2_-treated cells when compared to control (two right columns). Upper or lower extreme red boxes represent the biggest and smallest values of the data set. Lower extreme, the lowest or smallest value in a set of data. Blue boxes represent the median distribution of intensity values (c).

Using Agilent's bovine 4 × 44 K chip, we found a total of 17,114 hits in the microarray. Of these, 1127 were differentially expressed between the control group (PGE_2_ concentration = 0), and the rest of the doses used were considered together at a *P* value of 0.05 and 2x fold change. As a general trend, there were more genes downregulated (763 and 529 at *P* < 0.05 and *P* < 0.01, resp.) than upregulated (364 and 86, resp.). The FDR was of 0.05%. Due to the smaller differences in the number of deregulated genes when the comparison was made among different doses (data not shown), further detailed analysis was performed only among nonprimed cells (dose 0) and primed cells (doses 1, 2, and 3). As a result, the top 40 most deregulated genes were selected (Table 3) and subjected to bioinformatic analysis for gene ontology and network interactions. In the cat whisker plot assay, the two cell lines and the three different doses were considered. Apparently, there is no effect of the cell line on the results of the plot (Figure 5).

#### 3.2.2. Gene Ontology and Network Interactions

**Figure 6:**
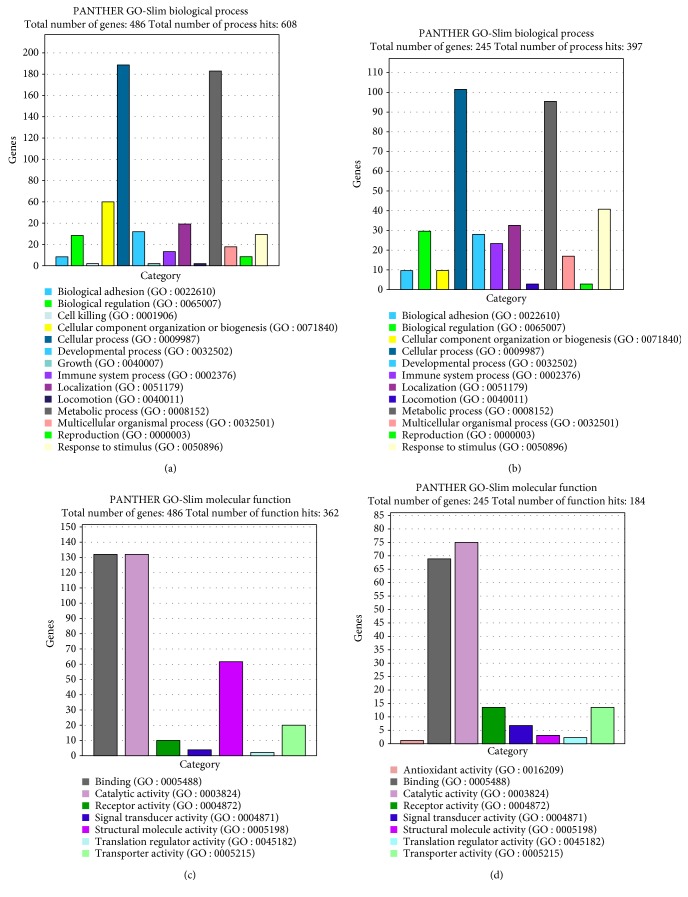
Gene ontology analysis of the main biological processes and molecular functions represented by the upregulated genes in 0 versus all doses of PGE_2_ (a, c) and downregulated genes in 0 versus all doses of PGE_2_ (b, d), respectively.

When the GO analysis was performed, the most affected biological processes were cellular component organization or biogenesis and cellular and metabolic processes. Among other represented processes found were biological regulation, development, growth, and immune system. The most represented molecular functions were binding, catalytic, receptor, transport, and structural molecule activity. The pathways represented with more hits among the upregulated genes were angiogenesis, B- and T-cell activation, blood coagulation, endothelin signaling, gonadotropin-releasing hormone receptor, heterotrimeric G-protein receptor, inflammation mediated by chemokine and cytokine signaling, and PDGF and Wnt signaling. For the downregulated genes, the pathways with the most hits were angiogenesis, cadherin signaling, gonadotropin-releasing hormone receptor, Huntington disease, inflammation mediated by chemokine and cytokine signaling, integrin signaling, and Wnt and TGF-beta signaling (Figure 6).

**Figure 7:**
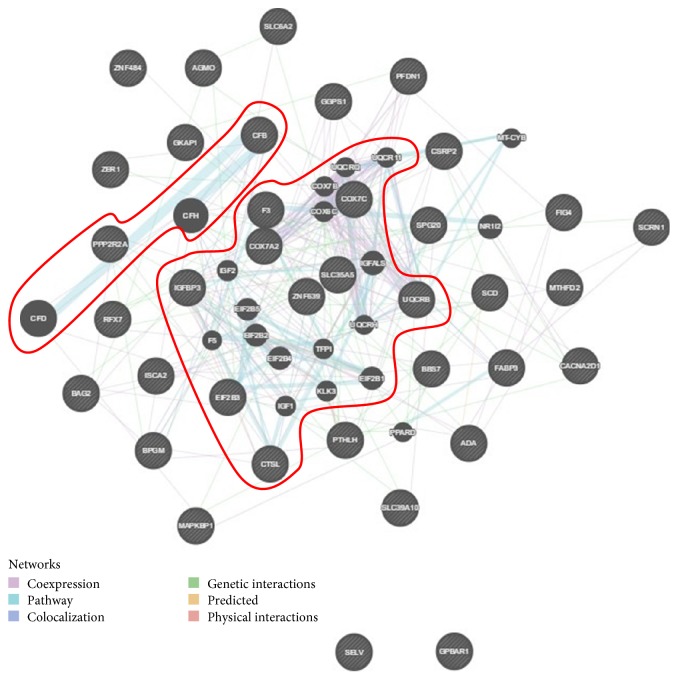
Readout of GeneMania Prediction Server showing gene interaction networks for the top 38 most differentially expressed genes. Of the top 40 differentially expressed genes, only LOC781494 and SNHG3 gene symbols were not recognized by the software. The colors of connections suggest the type of interaction as listed in the legend. (For interpretation of the references to color in this figure legend, the reader is referred to the Web version of this article.) Englobed in red are centrally located (more interacting genes) and a set of four genes peripherally located, but with a strong interaction.

GeneMania Prediction Server was used to determine the potential interactions among the top 40 most differentially expressed genes. From all the genes analyzed, only two (LOC781494 and SNHG3) were unrecognized by the program and 38 of 40 queried genes were connected. The predominant interaction was coexpression (70.5%) (Figure 7). For a more detailed mining analysis, we divided the global network into three fractions: (1) centrally and (2) peripherally located genes (*n* = 10 and *n* = 24, resp.) and (3) genes neither centrally nor peripherally located but with strong interactions between them (*n* = 4), aiming to examine the actual role of a given deregulated gene in its network of interactions. In the first fraction of centrally located genes, the network of interactions is wider and stronger, thus indicating a pivotal role of these genes in the complex response to PGE_2_ challenge. Five genes were upregulated and five were downregulated in this category. There are two major pathways appeared to be affected in this group of interacting genes; one is the upregulation of the COX7 superfamily and its related pathways such as the AMPK enzyme complex pathway and respiratory electron transport, ATP synthesis by chemiosmosis coupling, and heat production by uncoupling proteins; GO annotations related to this gene include electron carrier activity and cytochrome c oxidase activity; another is the downregulation of the insulin-like growth factor binding protein and its related pathways which include myometrial relaxation and contraction pathways and development of IGF-1 receptor signaling; GO annotations related to this gene include fibronectin binding and insulin-like growth factor I binding.

**Figure 8:**
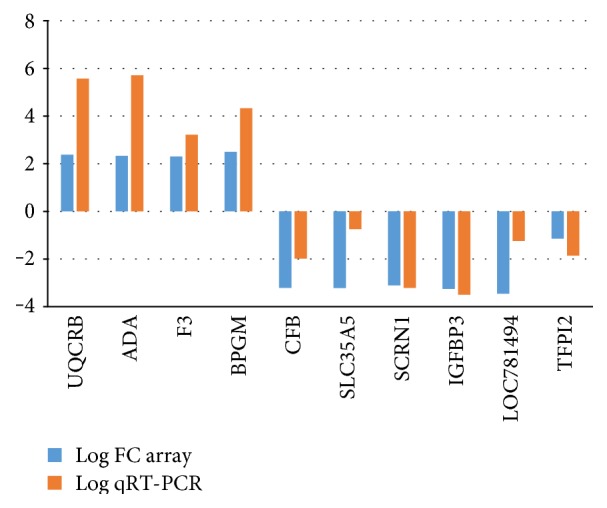
Validation of the microarray using relative expression of the selected genes by quantitative real-time polymerase chain reaction (qRT-PCR). Control embryos were used as calibrator to determine the fold change in the qRT-PCR. B-actin was used as housekeeping genes in all qRT-PCRs. In the qRT-PCR, all genes had the same expression pattern as in the microarray experiment. There is a linear correlation between data from qRT-PCR and microarray (*r* = 0.89, *P* < 0.05). The three equally expressed genes mentioned in the text (BAX2, COX2, and IL-1) were not plotted in this figure.

In order to validate the microarray findings, a qPCR was performed on 13 selected genes, and there was an absolute coincidence between the microarray and qPCR data, with an average value of *R* = 0.89 (Figure 8).

## 4. Discussion

During the partum and immediately after it, there is a high risk for the uterus to be infected by pathogens, mainly bacteria; these infections can impinge upon appropriate uterine involution and repopulation and upon return to cyclicity [25]. It has been postulated that such repopulation is mediated by resident or migrating MSCs [3]. In this study, we successfully isolated and characterized cells with characteristics of MSCs from the endometrium of cows during the postpartum of healthy cows, as well as from cows with subclinical and clinical endometritis. The cells used in this work cannot strictly be called pure MSCs. There are no specific MSC markers that allow identification of pure MSC; in fact, most likely, they are heterogeneous and nonclonal cultures of mesenchymal stromal cells which contain a subpopulation of stem cells with different multipotential properties, committed progenitors, and differentiated cells [26]. Nevertheless, the unique properties of the cells of this research, including their multilineage differentiation potential, are their ready availability and their extensive capacity for in vitro expansion indicating indeed the presence of MSCs among the mixed population used. As mentioned above, there are no clear markers of adult stem cells. Only in humans, the minimal criteria for such markers have been set, but not for other species including the bovine [27]. However, the surface phenotype, in conjunction with other functional criteria, best identifies MSC. These criteria, however, apply only to human MSC. For other species, particularly for farm animals, expression of surface antigens is not universally well characterized and the recommended markers may not apply to nonhuman systems.

Previously, our group has reported similar cells in cyclic cows [19, 20]; therefore, it was not unexpected to identify equivalent cell populations in puerperal healthy endometrium. To a similar extent, cells displaying the same properties were identified in this study in cows with subclinical endometritis, and CE and CDT were not different from healthy cows. All the primary cell cultures derived here displayed a fibroblast-like morphology with plastic adherence. Moreover, when the cells were seeded at low density, all the primary cultures yielded colonies; nonetheless, the cloning efficiency was higher in cells isolated from healthy cows, as was the cell doubling time. In clinically ill cows, the situation was different; clonogenic efficiency of 0.08 ± 0.1% and CDT of 41.97 ± 1.4 hours were found. Similar low values of clonogenic efficiency (0.02%) have been reported in human endometrial stromal cells [28, 29]. Additionally, in porcine endometrial MSCs, values of up to 0.035% have been recorded [16]. Although there was a low clonogenic efficiency of cells derived from cows with clinical endometritis, it was demonstrated that all cells were able to form colonies during bovine postpartum. This finding supports the idea of the existence of putative niches of MSCs present in the endometrium [3]. In addition, a longer doubling time than what was observed in this study has been described for human basal decidua stem cells with a value of 2.21 ± 0.21 days [30].

It seems that endometritis forces cells to grow slower and to have a lower cloning efficiency; all of these may be indicative of a reduced cell viability or senescence. Although not included in the results section, we found that cells coming from endometritis-ill animals in general, particularly from clinical endometritis, tended to detach from the culture vessels and were harder to culture *in vitro*. This may be related to inflammation, since no contamination with bacteria, fungi, or yeast was ever found in these or the other cells. Inflammation modifies the physical properties of cell membranes causing severe tissue damage and/or cellular necrosis, thus affecting the tissue's own regenerative capacity during postpartum [31]. It is believed that inflammation can affect progenitor stem cells directly in the total number of cell divisions and cause more premature aging, evidencing slower growth and affecting the outcome of cell differentiation [32].

We further detected that upon induction to trilineage differentiation, only the isolated endometrial cells from cows with clinical endometritis did not differentiate to adipogenic lineage. The adipogenic differentiation in the endometrial cells of healthy cows cycling has been reported previously in our and other research groups [18–20, 33]. The cells of the healthy endometrium and subclinical endometritis during postpartum endometrium have typical functional characteristics described for MSCs, such as fibroblast-like morphology with plastic adherence, high proliferative capacity, clone formation, and the ability to differentiate into chondrogenic, osteogenic, and adipogenic lineages *in vitro* [34]. This does not seem to be the case for cells derived from cows with clinical endometritis. This probably reflects a more differentiated status of cells or alternatively, that different and more committed progenitor cells are present. A similar phenomenon was found for endometrial cells of healthy cycling cows during the early luteal phase [19]. The presence of several subsets of stem cells with more mature progeny in the tissue limits the capacity of cellular self-renewal and alters the differentiation potential [35, 36]. It is presented in the literature that infection and inflammation can inhibit the regeneration of traumatized endometrium by effector molecules, which generate damage on resident cells responsible for tissue repair and regeneration [37].

It cannot be excluded that cells obtained from animals with endometritis in our study entered into senescence or at least displayed some features of senescent cells, although we did not study these markers in our research. Propagation of primary cells in vitro is often hampered by senescence in experimental models; this seems to be the case for MSCs as well. In rat MSCs, senescence correlated well with downregulation of genes involved in stem cell maintenance and DNA damage repair genes, as well with a decrease in differentiation potential, particularly the adipogenic potential [38]. In these experiments, senescent cells markedly upregulated genes involved in remodeling of extracellular matrix or in mediating local inflammation. Others, using MSCs from Rett syndrome patient showed precocious signs of senescence in comparison with the MSCs of healthy-patient control groups [39]. Authors also detected the downregulation of several stemness genes such as OCT4 and NANOG, concurrent with upregulation of lineage-specific genes, such as those involved in osteogenesis.

There are no defined gene markers for endometrial MSCs in cattle or species other than humans [27]. In this study, we detected the expression of two surface markers of MSCs, CD44, and CD117. While the former might be present in contaminating mature fibroblast populations [40], the latter has not been reported in such cells. In the bovine, CD117 is expressed in both myometrial and endometrial epithelial and stromal cell cultures; this is indicative of an ancestral bone marrow precursor, which probably migrates to the uterus throughout the animal's life and occurs regardless of its age or ovarian hormonal status [33]. We previously identified this gene also expressed in cattle endometrial MSCs regardless of the stage of the estrus cycle, and we propose it as a surface marker of cattle endometrial MSCs [19, 20]. CD44 has been detected in the endometrial MSCs of some farm animal species such as porcine, ovine, and bovine [16, 17, 19, 41] and in mares (Cabezas et al., unpublished).

Nuclear pluripotency markers OCT4 and SOX2, but not NANOG, were also found at both gene and protein in samples, which is coincident with our previous findings in cattle [19, 20] and the finding of others in the uterus of healthy and diseased women [42–44], as well as in the uterus of cows [16] and in cultured stromal cells of porcine endometrium [45]. The expression of stemness markers decreased in cultured cells in comparison to their presence in the uterine biopsies. It is suggested that adhesion to plastic and *in vitro* culture generates a loss of characteristics and cell markers, and this has been observed in adipose-derived adherent stromal cells [46] and in cattle endometrial cells derived from follicular phase of the estrus cycle [20]. The presence of pluripotency markers together with differentiation markers may indicate that endometrial stem cells might come from residual fetal stem cells bonded in various organs [47] or point to the existence of several stem cell populations in the bovine endometrium, which vary in their functional properties according to the inflammatory state of bovine endometrium during the postpartum period.

Information regarding the link between intrinsic inflammatory and regenerative pathways in endometritis is scarce. We propose that inflammation during endometritis triggers PGE_2_ secretion and that cellular changes in the endometrial tissue in response to endometritis involve PGE_2_-mediated activation of resident stromal progenitor/stem cells. We did not attempt to quantify local uterine PGE_2_ secretion, since it has been proven troublesome and inaccurate [11, 48] and blood determination of PGE_2_ does not necessarily reflect the actual endometrial levels. Previously, we showed that endometrial MSCs at the late luteal phase do respond to PGE_2_ administration creating an autocrine-paracrine-positive feedback leading to more PGE_2_ secretion (unpublished data). The rationale to using a priming approach as described here was to minimize the bias of distinct PGE_2_ expression levels that can exist in cells derived from cattle with endometritis; thus, we decided to use a cell model which had been previously tested in the laboratory, with two main attributes: (1) cells do not secrete basal levels of PGE_2_ and (2) they are responsive to it upon challenge. We found 1127 genes that were differentially expressed between dose 0 and the rest of the doses of PGE_2_, and we found subtle differences when doses of PGE_2_ were compared among their selves.

The analysis of gene interaction networks showed that there was an upregulation of the COX7 family. This may imply a shift in the production of prostaglandins as end products of the arachidonic acid pathway which is involved in COX2 synthesis and ultimately in PGE_2_ release and action. It is vastly supported by the literature that PGE_2_ upregulates its own secretion via COX2 and related pathways [49]; therefore, it is tempting to propose that challenging cells with PGE_2_ lead to a positive feedback regulation of its precursor. The downregulation of the IGFBP pathway and consequently of the IGF-1 receptor signaling may be correlated with the slow proliferating phenotype of the endometrial cells under endometritis conditions. It is possible that the collaborative action between these two pathways leads to an overexpression of PGE_2_ and to the slowing down of cell growth. This situation may occur in the environment of the uterus under endometritis where high levels of PGE_2_ are present. Finally, we found a very robust interaction between a precise set of four genes located peripherally in the gene interaction netwok map, but keeping a strong, precise interaction among them. Three of these genes are of the complement factor family (CF family genes B, D, and H) and the PPARD (peroxisome proliferator-activated receptor delta). The former genes are involved in immune response, induced complement pathway, and *Staphylococcus aureus* infection. Downregulation in these genes underlies complement factor D deficiency, which is associated with recurrent bacterial meningitis infections in human patients [50]. The latter gene is involved in Wnt signaling pathway (WikiPathways).

Activation of the PGE_2_ pathway in the endometrium may lead to a shift in the proportion of normal stem cells that can be potentially identified and isolated. It has been reported that PGE_2_ during the follicular phase stimulates the growth of endometrial cells through the Wnt/*β*-catenin pathway, which in the case of porcine endometrium governs the cyclical cell regeneration process [51].

The GO data shown above are informative and describe general processes or functions, but a detailed look was taken at particular pathways affected in the deregulated genes. In that sense, the pathways represented with the most hits among the upregulated genes were angiogenesis, B- and T-cell activation, blood coagulation, endothelin signaling, gonadotropin-releasing hormone receptor, heterotrimeric G-protein receptor, inflammation mediated by chemokine and cytokine signaling, and PDGF and Wnt signaling. For the downregulated genes, the pathways with more hits were angiogenesis, cadherin signaling, gonadotropin-releasing hormone receptor, Huntington's disease, inflammation mediated by chemokine and cytokine signaling, integrin signaling, Wnt *β*-catenin, and TGF-*β* signaling.

In summary, we were able to demonstrate that challenging endometrial MSCs with PGE_2_ lead to a massive rearrangement of the cell transcriptome profile, which to the best of our knowledge has not been previously reported in the literature. The most remarkable genes and pathways that are affected are related to immune response, angiogenesis, and cell proliferation. The addition of PGE_2_ to endometrial cells seemed to downregulate the cell immune response. This is in agreement with our findings for adipose-derived horse MSCs (unpublished). No data were available in the literature as compared with our findings of downregulation of immune response in the endometrium. This however makes sense, since MSCs are shown in the literature and our own unpublished research to be immunoprivileged upon stimulation with proinflammatory licensing molecules such as INF gamma [52].

In our experiments, the addition of PGE_2_ to endometrial cells leads to a downregulation of Wnt-*β*-catenin and TGF-*β* signaling, which was unexpected. Bayne et al. [53] demonstrated that PGE_2_ regulates germ cell function in vivo during ovarian development in humans, acting upon mediators such as activin A and others to affect the expression of pluripotency markers in the first trimester fetal human ovaries, which includes E2 and E4 receptor and VASA and DAZL genes, but not OCT4. Goessling et al. [54] using *in vitro* and *in vivo* mouse (and zebra fish) models unequivocally showed that PGE_2_ interacts with the Wnt pathway by direct phosphorylation of *β*-catenin and served as a master regulator of stem cell recruitment and organ regeneration in vertebrates. The Wnt/*β*-catenin pathway stimulated by PGE_2_ is the same canonical stem cell recruitment path used by HSC cells and embryonic stem cells [54]. Most likely, brief exposure to PGE_2_ locally induces tissue regeneration via local stem cell recruitment or from migrating hematopoietic stem cells, while prolonged exposition could potentially lead to chronic inflammation such as persistent endometriosis. Although the studies mentioned above did not target stem cell regeneration of the uterine endometrium, it is tempting to speculate that a similar process might operate in postmenstrual endometrial repopulation in women and in cyclic regeneration of the endometrium in ruminants or in PGE_2_-mediated endometritis. Our findings associated with these two pathways in the *in vitro* model used are in concordance with the observed limited availability and functionality of putative MSC isolated from cows with subclinical or clinical endometritis in our research.

As discussed above, we cannot rule out the possibility that our cells entered into senescence. It is sufficiently demonstrated in the literature that PGE_2_ may trigger the onset of senescence. Exposure *in vitro* and *in vivo* to PGE_2_ may lead to phenotypes of senescence [55, 56]. When CD8+ T cells were exposed to PGE_2_, the cells displayed markers of senescence such as loss of CD28 expression, reduced telomerase activity linked to telomere shortening, and overexpression of p16, COX2, and intracellular cAMP [55]. These data suggest that increased PGE_2_ may contribute to the development of senescence of immune cells. Likewise, it is described that PGE_2_ acted in an autocrine loop through EP receptors inducing high COX2 levels and senescence in human fibroblasts via an independent ROS and a dependent PGE_2_/EP intracrine pathway [56].

In the microarray data of our research, we were not able to identify differential regulation of key senescence markers, including CD28, p16, telomerase, and others discussed above. Of interest, a Rho GTPase-activating protein 27 (ARHGAP27) was downregulated and p27, a marker of senescent cell expression, was not detected. We believe that at least in our experimental conditions, we found that exposure of putative MSCs *in vitro* to PGE_2_ did not induce senescence, although chronical exposure *in vivo* during endometritis to PGE_2_-induced senescence in the cells derived from endometrium was not addressed here and cannot be ruled out.

## 5. Conclusion

We confirmed the presence of progenitor MSCs in bovine endometrium during the postpartum period. The pathological inflammation of the endometrium modifies and limits some functional characteristics of the MSCs. This becomes more evident in clinical endometritis than in subclinical, suggesting the presence of a more differentiated progeny of cells. It is possible that tissue damage generated by inflammation may directly affect the cellular niche and/or promote cell proliferation, in order to restore tissue homeostasis. The exposure *in vitro* of MSCs to a mediator of inflammation such as PGE_2_ modifies their transcriptomic profile, covering mainly biological processes such as cellular component organization or biogenesis and cellular and metabolic processes, such as biological regulation, development, growth, and immune system. Thus, PGE_2_ may have a potential role in the fate of stem cell activation, migration, homing processes during pathological, uterine inflammation such as in endometritis, and also in the healthy puerperal endometrium.

## Figures and Tables

**Table 1:** Details of primers used for qRT-PCR analysis of selected genes used in the PCR experiments.

Gene name	Primer sequences	Annealing *T* (°C)	GenBank accession number
OCT4	F: 5′-GGAGAGCATGTTCCTGCAGTGC 3′ R: 5′-ACACTCGGACCACGTCCTTCTC 3′	58°C	NM_174580
NANOG	F: 5′-TTCCCTCCTCCATGGATCTG 3′ R: 5′-ATTTGCTGGAGACTGAGGTA 3′	58°C	NM_001025344
SOX2	F: 5′-CGAGTGGAAACTTTTGTCCG 3′ R: 5′-GGTATTTATAATCCGGGTGTT 3′	55°C	NM_001105463
CD44	F: 5′-GACTGTACATCGGTCACGGACC 3′ R: 5′-GGTATAACGGGTGCCATCACGG 3′	58°C	S63418.1
CD117	F: 5′-TCCAAAACTCATCTGTCTCACC 3′ R: 5′-CCCACATCGTTATAAGCCCTG 3′	58°C	AF263827.1
ACTB	F: 5′-GGCCAACCGTGAGAAGATGACC 3′ R: 5′-GAGGCATACAGGGACAGCACAG 3′	58°C	BT030480.1
UQCRB	F: 5′-CCTTTATAATGACAGAGTGTTTCGC 3′ R: 5′-ACGGTTCAAGGTAGGATTTATCC 3′	62°C	NM_001034797
ADA	F: 5′-GAGGAGCTACAGAACATCATCG 3′ R: 5′-AATCCTTTTGACAGCCTCCC 3′	62°C	NM_173887
F3	F: 5′-GAGTCCAGAAAGTCCCATCAAG 3′ R: 5′-TGATCACCAGCATCACTGTG 3′	62°C	NM_173878
BPGM	F: 5′-GTATTGCGTGGTAAAACCGTTC 3′ R: 5′-CCCCAGTAGGAAGAGTAATGTTG 3′	62°C	NM_001035402
CFB	F: 5′-GGGTGCTATTGTGTCTGAGTAC 3′ R: 5′-CTTTTACCTCCCACTCCTTCC 3′	62°C	NM_001040526
SLC35A5	F: 5′-CTCAAGTCGCATCCTACTGG 3′ R: 5′-GACACAAGCGCACAGAAAAC 3′	62°C	NM_001076025
SCRN1	F: 5′-ATCATGAACCTGAGAGCAAGG 3′ R: 5′-AGGTCTGCTTATCACAATGGC 3′	62°C	NM_001110803
IGFBP3	F: 5′-CGCTACAAGGTTGACTACGAG 3′ R: 5′-GTTCAGCGTGTCTTCCATTTC 3′	62°C	NM_174556
LOC781494	F: 5′-AAGATTCTGGAGAGTGCTGTG 3′ R: 5′-GACGAAGCAGATGGAGTACAC 3′	62°C	NM_001101279
TFPI2	F: 5′-ACGTGTATGGACTTCTGTGC 3′ R: 5′-CCACACCCAGTATAGTTGAAGG 3′	58°C	NM_182788
BAX2	F: 5′-AGGGTTTCATCCAGGATCGAGC 3′ R: 5′-TCATCTCCGATGCGCTTCAGAC 3′	58°C	NM_001191220.1
COX2	F: 5′-GTCCCGTCCAGGCTTATATTAC 3′ R: 5′-GGACTAACTCAAGGACAATGGG 3′	62°C	DQ347624.1
IL-1	F: 5′-AAGGAGAATGTGGTGATGGTG 3′ R: 5′-TGTAATGTGCTGATCTGGGC 3′	62°C	NM_174092

**Table 2:** Clonal efficiency (CE) and cell doubling time (CDT) of fibroblast-like colony formation of cell derived from primary cultures *in vitro* of postpartum healthy endometrium (PPHE) with subclinical (PPSE) and clinical endometritis (PPCE). Different superscript letters in the same column indicate statistically significant differences. *P* value < 0.05. Each experiment was repeated three times for each cell line.

Cell line	Number of colonies	CE (%)	CDT (hrs)
PPHE1	6.3	0.75	29.8
PPHE2	4.7	0.56	28.4
PPHE3	5.7	0.67	31.2
PPHE4	4.5	0.52	28.9
PPHE5	5.9	0.71	31.7
PPHE ($\bar{x}$ ± S.D.)	5.42 ± 0.7^a^	0.64 ± 0.1^a^	30 ± 0.4^a^
PPSE1	4	0.48	33.9
PPSE2	4.3	0.52	35.2
PPSE3	3.7	0.45	34.2
PPSE4	3.9	0.55	34.9
PPSEl5	4.4	0.44	35.5
PPSE ($\bar{x}$ ± S.D.)	4.06 ± 0.3^ab^	0.49 ± 0.1^ab^	34.74 ± 0.7^ab^
PPCE1	1.5	0.16	42
PPCE2	1	0.08	40.6
PPCE3	0	0	43.3
PPCE ($\bar{x}$ ± S.D.)	0.83 ± 0.8^b^	0.08 ± 0.1^b^	41.97 ± 1.4^b^

**Table 3:** Top 40 differentially expressed genes (DEGs) between doses 0 and doses 1, 2, and 3 of PGE_2_ in MSC cells isolated from late luteal phase of estrus cycle in cattle.

Gene symbol	GenBank accesion	Gene name	Fold change	Regulation
LOC781494	A_73_P048499	*Bos taurus* myeloid-associated differentiation marker-like	−11.00	Down
ZNF484	A_73_P086786	*Bos taurus* zinc finger protein 484	−10.96	Down
CFB	A_73_118840	*Bos taurus* complement factor B	−9.73	Down
SPG20	A_73_P077131	*Bos taurus* spastic paraplegia 20 (Troyer syndrome)	−9.69	Down
IGFBP3	A_73_120953	*Bos taurus* insulin-like growth factor binding protein 3	−9.53	Down
SLC35A5	A_73_P482423	*Bos taurus* solute carrier family 35, member A5	−9.31	Down
SLC39A10	A_73_P094756	*Bos taurus* solute carrier family 39	−9.13	Down
SCRN1	A_73_P043336	*Bos taurus* secernin 1	−8.61	Down
AGMO	A_73_113510	*Bos taurus* alkylglycerol monooxygenase	−8.54	Down
CTSL1	A_73_113548	*Bos taurus* cathepsin L1	−8.47	Down
ZNF639	A_73_P107146	*Bos taurus* zinc finger protein 639	−8.37	Down
CACNA2D1	A_73_P421251	*Bos taurus* calcium channel, voltage-dependent, alpha 2/delta subunit 1	−7.60	Down
GGPS1	A_73_P424671	*Bos taurus* geranylgeranyl diphosphate synthase 1	−7.58	Down
FIG4	A_73_P257991	*Bos taurus* FIG4 homolog	−6.76	Down
MAPKBP1	A_73_P383151	*Bos taurus* mitogen-activated protein kinase binding protein 1	−6.49	Down
GKAP1	A_73_P417031	*Bos taurus* G kinase anchoring protein 1	−6.40	Down
RFX7	A_73_110155	*Bos taurus* regulatory factor X, 7	−6.40	Down
PPP2R2A	A_73_P086641	*Bos taurus* protein phosphatase 2, regulatory subunit B, alpha	−6.27	Down
BBS7	A_73_P087146	*Bos taurus* Bardet-Biedl syndrome 7	−6.07	Down
ZER1	A_73_P083316	*Bos taurus* zer-1 homolog (C, elegans)	−5.97	Down
COX7C	A_73_P064916	*Bos taurus* cytochrome c oxidase subunit VIIc	4.59	Up
FABP3	A_73_P378211	*Bos taurus* fatty acid binding protein 3	4.60	Up
MTHFD2	A_73_115733	*Bos taurus* methylenetetrahydrofolate dehydrogenase	4.62	Up
COX7A2	A_73_P087911	*Bos taurus* cytochrome c oxidase subunit VIIa polypeptide 2	4.64	Up
EIF2B3	A_73_P257151	*Bos taurus* eukaryotic translation initiation factor 2B, subunit 3	4.67	Up
F3	A_73_109579	*Bos taurus* coagulation factor III	4.90	Up
BAG2	A_73_P275196	*Bos taurus* BCL2-associated athanogene 2	4.91	Up
ADA	A_73_102071	*Bos taurus* adenosine deaminase	5.02	Up
SNHG3	A_73_120992	*Bos taurus* small nucleolar RNA host gene 3	5.09	Up
UQCRB	A_73_P494163	*Bos taurus* ubiquinol-cytochrome c reductase binding protein	5.17	Up
SCD	A_73_106141	*Bos taurus* stearoyl-CoA desaturase (delta-9-desaturase)	5.17	Up
PFDN1	A_73_103325	*Bos taurus* prefoldin subunit 1	5.48	Up
SLC6A2	A_73_P066991	*Bos taurus* noradrenaline transporter	5.63	Up
BPGM	A_73_P047401	*Bos taurus* 2,3-bisphosphoglycerate mutase	5.63	Up
GPBAR1	A_73_105396	*Bos taurus* G protein-coupled bile acid receptor 1	5.71	Up
ISCA2	A_73_P040806	*Bos taurus* iron-sulfur cluster assembly 2 homologs	5.76	Up
CSRP2	A_73_118163	*Bos taurus* cysteine and glycine-rich protein 2	5.91	Up
COX7C	A_73_P080311	*Bos taurus* cytochrome c oxidase subunit VIIc	6.74	Up
PTHLH	A_73_P038881	*Bos taurus* parathyroid hormone-like hormone	6.83	Up
SELV	A_73_P067596	*Bos taurus* selenoprotein V	7.63	Up

## References

[1] Arai M., Yoshioka S., Tasaki Y., Okuda K. (2013). Remodeling of bovine endometrium throughout the estrous cycle. *Animal Reproduction Science*.

[2] Mitko K., Ulbrich S. E., Wenigerkind H. (2008). Dynamic changes in messenger RNA profiles of bovine endometrium during the oestrous cycle. *Reproduction*.

[3] Chan R. W. S. (2004). Clonogenicity of human endometrial epithelial and stromal cells. *Biology of Reproduction*.

[4] Cao M., Chan R. W., Yeung W. S. (2015). Label-retaining stromal cells in mouse endometrium awaken for expansion and repair after parturition. *Stem Cells and Development*.

[5] Okano A., Tomizuka T. (1996). Post partum uterine involution in the cow. *Theriogenology*.

[6] Healy L. L., Cronin J. G., Sheldon I. M. (2014). Endometrial cells sense and react to tissue damage during infection of the bovine endometrium via interleukin 1. *Scientific Reports*.

[7] Sheldon I. M., Williams E. J., Miller A. N. A., Nash D. M., Herath S. (2008). Uterine diseases in cattle after parturition. *The Veterinary Journal*.

[8] Herath S., Lilly S. T., Santos N. R. (2009). Expression of genes associated with immunity in the endometrium of cattle with disparate postpartum uterine disease and fertility. *Reproductive Biology and Endocrinology*.

[9] Földi J., Kulcsár M., Pécsi A. (2006). Bacterial complications of postpartum uterine involution in cattle. *Animal Reproduction Science*.

[10] Nakanishi M., Rosenberg D. W. (2013). Multifaceted roles of PGE_2_ in inflammation and cancer. *Seminars in Immunopathology*.

[11] Barański W., Łukasik K., Skarzyński D., Sztachańska M., Zduńczyk S., Janowski T. (2013). Secretion of prostaglandins and leukotrienes by endometrial cells in cows with subclinical and clinical endometritis. *Theriogenology*.

[12] Mateus L., Lopes da Costa L., Diniz P., Ziecik A. J. (2003). Relationship between endotoxin and prostaglandin (PGE_2_ and PGFM) concentrations and ovarian function in dairy cows with puerperal endometritis. *Animal Reproduction Science*.

[13] Hoggatt J., Singh P., Sampath J., Pelus L. M. (2015). Prostaglandin E2 enhances hematopoietic stem cell homing, survival, and proliferation. *Blood*.

[14] Cervelló I., Martínez-Conejero J. A., Horcajadas J. A., Pellicer A., Simón C. (2007). Identification, characterization and co-localization of label-retaining cell population in mouse endometrium with typical undifferentiated markers. *Human Reproduction*.

[15] Schwab K. E., Gargett C. E. (2007). Co-expression of two perivascular cell markers isolates mesenchymal stem-like cells from human endometrium. *Human Reproduction*.

[16] Miernik K., Karasinski J. (2012). Porcine uterus contains a population of mesenchymal stem cells. *Reproduction*.

[17] Letouzey V., Tan K. S., Deane J. A., Ulrich D. (2015). Isolation and characterisation of mesenchymal stem/stromal cells in the ovine endometrium. *PLoS One*.

[18] Mehrabani D., Rahmanifar F., Mellinejad M. (2015). Isolation, culture, characterization, and adipogenic differentiation of heifer endometrial mesenchymal stem cells. *Comparative Clinical Pathology*.

[19] Cabezas J., Lara E., Pacha P. (2014). The endometrium of cycling cows contains populations of putative mesenchymal progenitor cells. *Reproduction in Domestic Animals*.

[20] Lara E., Rivera N., Rojas D., Rodríguez-Alvarez L. L., Castro F. O. (2017). Characterization of mesenchymal stem cells in bovine endometrium during follicular phase of oestrous cycle. *Reproduction in Domestic Animals*.

[21] LeBlanc S. J., Duffield T. F., Leslie K. E. (2002). Defining and diagnosing postpartum clinical endometritis and its impact on reproductive performance in dairy cows. *Journal of Dairy Science*.

[22] Sheldon I. M., Lewis G. S., LeBlanc S., Gilbert R. O. (2006). Defining postpartum uterine disease in cattle. *Theriogenology*.

[23] Vidal M. A., Kilroy G. E., Johnson J. R., Lopez M. J., Moore R. M., Gimble J. M. (2006). Cell growth characteristics and differentiation frequency of adherent equine bone marrow–derived mesenchymal stromal cells: adipogenic and osteogenic capacity. *Veterinary Surgery*.

[24] Warde-Farley D., Donaldson S. L., Comes O. (2010). The GeneMANIA prediction server: biological network integration for gene prioritization and predicting gene function. *Nucleic Acids Research*.

[25] Sheldon I. M., Dobson H. (2004). Postpartum uterine health in cattle. *Animal Reproduction Science*.

[26] Squillaro T., Peluso G., Galderisi U. (2016). Clinical trials with mesenchymal stem cells: an update. *Cell Transplantation*.

[27] Dominici M., Le Blanc K., Mueller I. (2006). Minimal criteria for defining multipotent mesenchymal stromal cells. The International Society for Cellular Therapy position statement. *Cytotherapy*.

[28] Schwab K. E., Hons B. B. S., Wah R. (2005). Putative stem cell activity of human endometrial epithelial and stromal cells during the menstrual cycle. *Fertility and Sterility*.

[29] Gargett C. E. (2006). Identification and characterisation of human endometrial stem/progenitor cells. *Australian and New Zealand Journal of Obstetrics and Gynaecology*.

[30] Lu G. H., Zhang S. Z., Chen Q. (2011). Isolation and multipotent differentiation of human decidua basalis-derived mesenchymal stem cells. *Journal of Southern Medical University*.

[31] Carneiro L. C., Cronin J. G., Sheldon I. M. (2016). Mechanisms linking bacterial infections of the bovine endometrium to disease and infertility. *Reproductive Biology*.

[32] Alongi D. J., Yamaza T., Song Y. (2010). Stem/progenitor cells from inflamed human dental pulp retain tissue regeneration potential. *Regenerative Medicine*.

[33] Łupicka M., Bodek G., Shpigel N., Elnekave E., Korzekwa A. J. (2015). Identification of pluripotent cells in bovine uterus: *in situ* and *in vitro* studies. *Reproduction*.

[34] Gargett C. E. (2007). Uterine stem cells: what is the evidence?. *Human Reproduction Update*.

[35] Weissman I. L. (2000). Stem cells: units of development, units of regeneration, and units in evolution. *Cell*.

[36] Sethe S., Scutt A., Stolzing A. (2006). Aging of mesenchymal stem cells. *Ageing Research Reviews*.

[37] Gargett C. E., Ye L. (2012). Endometrial reconstruction from stem cells. *Fertility and Sterility*.

[38] Galderisi U., Helmbold H., Squillaro T. (2009). In vitro senescence of rat mesenchymal stem cells is accompanied by downregulation of stemness-related and DNA damage repair genes. *Stem Cells and Development*.

[39] Squillaro T., Hayek G., Farina E., Cipollaro M., Renieri A., Galderisi U. (2008). A case report: bone marrow mesenchymal stem cells from a Rett syndrome patient are prone to senescence and show a lower degree of apoptosis. *Journal of Cellular Biochemistry*.

[40] Halfon S., Abramov N., Grinblat B., Ginis I. (2011). Markers distinguishing mesenchymal stem cells from fibroblasts are downregulated with passaging. *Stem Cells and Development*.

[41] de Moraes C. N., Maia L., Dias M. C. (2016). Bovine endometrial cells: a source of mesenchymal stem/progenitor cells. *Cell Biology International*.

[42] Matthai C., Horvat R., Noe M. (2006). Oct-4 expression in human endometrium. *Molecular Human Reproduction*.

[43] Ono M., Kajitani T., Uchida H. (2010). OCT4 expression in human uterine myometrial stem/progenitor cells. *Human Reproduction*.

[44] Pacchiarotti A., Caserta D., Sbracia M., Moscarini M. (2011). Expression of oct-4 and c-kit antigens in endometriosis. *Fertility and Sterility*.

[45] Bodek G., Bukowska J., Wisniewska J., Ziecik A. J. (2015). Evidence for the presence of stem/progenitor cells in porcine endometrium. *Molecular Reproduction and Development*.

[46] Yoshimura K., Shigeura T., Matsumoto D. (2006). Characterization of freshly isolated and cultured cells derived from the fatty and fluid portions of liposuction aspirates. *Journal of Cellular Physiology*.

[47] Ratajczak M. Z., Liu R., Ratajczak J., Kucia M., Shin D. M. (2011). The role of pluripotent embryonic-like stem cells residing in adult tissues in regeneration and longevity. *Differentiation*.

[48] Silva E., Gaivão M., Leitão S., Amaro A., Lopes da Costa L., Mateus L. (2008). Blood COX-2 and PGES gene transcription during the peripartum period of dairy cows with normal puerperium or with uterine infection. *Domestic Animal Endocrinology*.

[49] Sales K. J., Katz A. A., Davis M. (2001). Cyclooxygenase-2 expression and prostaglandin E2 synthesis are up-regulated in carcinomas of the cervix: a possible autocrine/paracrine regulation of neoplastic cell function via EP2/EP4 receptors. *The Journal of Clinical Endocrinology and Metabolism*.

[50] White R. T., Damm D., Hancock N. (1992). Human adipsin is identical to complement factor D and is expressed at high levels in adipose tissue. *The Journal of Biological Chemistry*.

[51] Bukowska J., Ziecik A. J., Laguna J., Gawronska-Kozak B., Bodek G. (2015). The importance of the canonical Wnt signaling pathway in the porcine endometrial stromal stem/progenitor cells: implications for regeneration. *Stem Cells and Development*.

[52] Krampera M. (2011). Mesenchymal stromal cell ‘licensing’: a multistep process. *Leukemia*.

[53] Bayne R. A., Eddie S. L., Collins C. S., Childs A. J., Jabbour H. N., Anderson R. A. (2009). Prostaglandin E2 as a regulator of germ cells during ovarian development. *The Journal of Clinical Endocrinology and Metabolism*.

[54] Goessling W., North T. E., Loewer S. (2009). Genetic interaction of PGE2 and Wnt signaling regulates developmental specification of stem cells and regeneration. *Cell*.

[55] Chou J. P., Ramirez C. M., Ryba D. M., Koduri M. P., Effros R. B. (2014). Prostaglandin E2 promotes features of replicative senescence in chronically activated human CD8+ T cells. *PLoS One*.

[56] Martien S., Pluquet O., Vercamer C. (2013). Cellular senescence involves an intracrine prostaglandin E2 pathway in human fibroblasts. *Biochimica et Biophysica Acta (BBA) - Molecular and Cell Biology of Lipids*.

